# Malaria incidence trends and their association with climatic variables in rural Gwanda, Zimbabwe, 2005–2015

**DOI:** 10.1186/s12936-017-2036-0

**Published:** 2017-09-30

**Authors:** Resign Gunda, Moses John Chimbari, Shepherd Shamu, Benn Sartorius, Samson Mukaratirwa

**Affiliations:** 1 School of Nursing and Public Health, University of KwaZulu-Natal, Durban, South Africa; 2 College of Health Sciences, University of KwaZulu-Natal, Howard Campus, Durban, South Africa; 3 Department of Community Medicine, University of Zimbabwe, Harare, Zimbabwe; 4 Discipline of Public Health Medicine, School of Nursing and Public Health, University of KwaZulu-Natal, Durban, South Africa; 5 School of Life Sciences, University of KwaZulu-Natal, Durban, South Africa

**Keywords:** Malaria, Incidence, Trends, Gwanda, Zimbabwe

## Abstract

**Background:**

Malaria is a public health problem in Zimbabwe. Although many studies have indicated that climate change may influence the distribution of malaria, there is paucity of information on its trends and association with climatic variables in Zimbabwe. To address this shortfall, the trends of malaria incidence and its interaction with climatic variables in rural Gwanda, Zimbabwe for the period January 2005 to April 2015 was assessed.

**Methods:**

Retrospective data analysis of reported cases of malaria in three selected Gwanda district rural wards (Buvuma, Ntalale and Selonga) was carried out. Data on malaria cases was collected from the district health information system and ward clinics while data on precipitation and temperature were obtained from the climate hazards group infrared precipitation with station data (CHIRPS) database and the moderate resolution imaging spectro-radiometer (MODIS) satellite data, respectively. Distributed lag non-linear models (DLNLM) were used to determine the temporal lagged association between monthly malaria incidence and monthly climatic variables.

**Results:**

There were 246 confirmed malaria cases in the three wards with a mean incidence of 0.16/1000 population/month. The majority of malaria cases (95%) occurred in the > 5 years age category. The results showed no correlation between trends of clinical malaria (unconfirmed) and confirmed malaria cases in all the three study wards. There was a significant association between malaria incidence and the climatic variables in Buvuma and Selonga wards at specific lag periods. In Ntalale ward, only precipitation (1- and 3-month lag) and mean temperature (1- and 2-month lag) were significantly associated with incidence at specific lag periods (p < 0.05). DLNM results suggest a key risk period in current month, based on key climatic conditions in the 1–4 month period prior.

**Conclusions:**

As the period of high malaria risk is associated with precipitation and temperature at 1–4 month prior in a seasonal cycle, intensifying malaria control activities over this period will likely contribute to lowering the seasonal malaria incidence.

## Background

Malaria is one of the most prevalent parasitic diseases worldwide [1]. There were approximately 214 million malaria cases and 438,000 malaria deaths globally in 2015, with most of these cases (88%) and deaths (90%) occurring in Africa [2]. Malaria contributes to a large public health burden with over 75% of the global clinical episodes due to *Plasmodium falciparum* concentrated in Africa [3]. Malaria is a public health problem in sub-Saharan Africa and is a leading cause of morbidity and mortality [4–6].

Zimbabwe experiences seasonal malaria transmission which is potentially epidemic [7]. Resistance of the malaria vectors to insecticides [8, 9] as well as parasite resistance to anti-malarial drugs [10] contribute to the challenges affecting control of malaria in Zimbabwe and regionally. The majority of malaria cases in Zimbabwe (98%) are caused by *P. falciparum* and the primary vector is *Anopheles arabiensis* [11]. *Plasmodium falciparum* is associated with severe and fatal disease. The risk for transmitting malaria in Zimbabwe is predominant during the rainy season (November to April).

The link between climate variability and vector-borne diseases has been established [12, 13]. Climate variability has the potential to either work for or against efforts to control the disease. Results from a study by Ebi et al. [14] suggested that changes in temperature and rainfall could alter the geographic distribution of malaria and it is expected that areas that were previously unsuitable for malaria transmission may become suitable in the future as a result of these changes in climate. The number of malaria infections can be influenced by climate factors, leading to increases or decreases in malaria incidence under climate variability [15].

Climatic factors such as rainfall and temperature play an important role in malaria transmission. Rainfall is responsible for creating mosquito breeding sites while temperature regulates the rate of development of the mosquito larvae and influences mosquito survival rates [16, 17]. Higher temperatures favour rapid multiplication of the *Plasmodium* inside the mosquito [18]. These two climatic variables create conditions that favour malaria transmission in endemic countries. Although it is well documented that malaria transmission is sensitive to climate, the predictions that have been made so far on the expansion of malaria endemic areas in relation to climate change seem to be mismatched across different areas [19] with the reduction in endemicity over time [18].

An understanding of how malaria incidences vary as a result of climate variability (present and recent past) is important for planning for future malaria control programmes [20]. Data on trends of diseases is needed to judge success of interventions to determine whether adjustments are required [21]. It also provides essential information on the changing malaria situation [20]. Information on the association between malaria incidence and lagged climatic conditions can be used in the development of malaria early warning systems. Most previous studies have assessed disease trends at global, regional or national levels [22–25]. In most cases, these studies were based on unconfirmed cases of malaria. In Zimbabwe, there is paucity of data on trends of malaria incidences at district and ward levels over longer periods (10 or more years). This is the first study to assess trends of a single disease at ward level in Zimbabwe using confirmed incident malaria. The objective of this study was to assess the trends of the incidences of malaria in rural Gwanda, Zimbabwe in relation to climatic variables.

## Methods

### Study area and population

The study was conducted in Gwanda District, the capital of Matabeleland South Province in southeast Zimbabwe. Malaria is one of the leading public health challenges in Gwanda district. The district accounts for the second highest number of recorded malaria cases in the province [26]. Malaria transmission unstable and is confined to the southern parts of the district [27]. The total population for the district is 137,461 people with 116,939 (85%) residing in rural areas. There are 26,773 households in rural areas with an average household size of 3.6 individuals [28]. Rural Gwanda has 24 wards (administrative unit within district). Gwanda lies in the natural region V which is characterized by very low rainfall resulting in frequent droughts in the area.

**Fig. 1:**
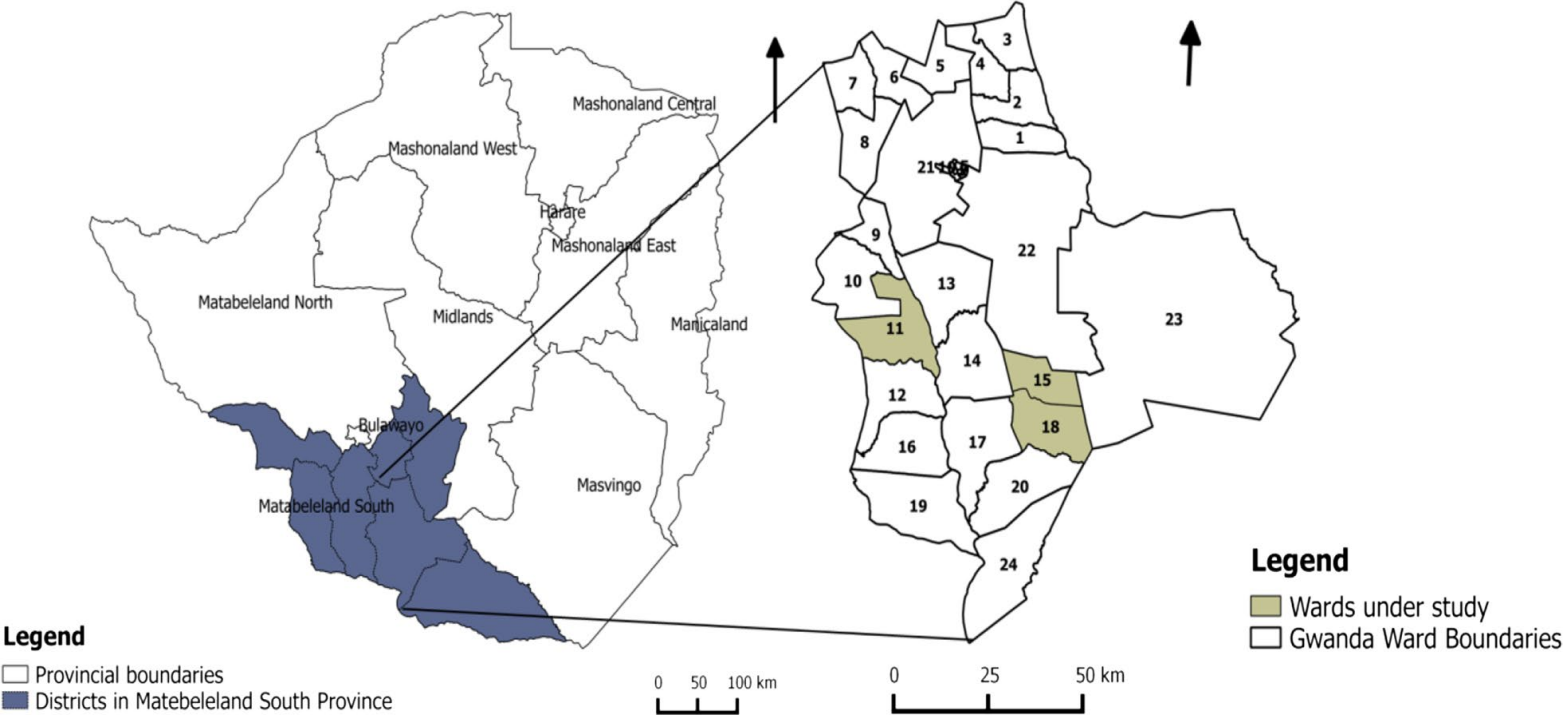
Map showing the three study wards of Buvuma (ward 15), Ntalale (ward 18) and Selonga (ward 11) in Gwanda district, Matabeleland south province, Zimbabwe

This study was conducted in three rural wards namely Buvuma, Ntalale and Selonga (Fig. 1) with a population of 5523, 4591 and 4697 people, respectively [28]. These three wards have similar socio-demographic characteristics and their communities have similar economic activities. The selected wards all have at least a dam and an irrigation scheme. The wards were purposively selected as they had the highest cumulative number of reported cases of malaria over the study period. There are 29 health facilities in the district including one provincial hospital and one mission hospital with the rest being clinics.

All suspected malaria cases are tested using a rapid diagnostic test (RDT). In addition to the RDT, a blood slides are prepared for each case and sent to the district laboratory for microscopic examination for parasites. Uncomplicated cases for malaria are treated with artemether and lumefantrine (Co-Artem^®^). The drug is administered according to body weight of the patient. Each malaria patient is reviewed after 3 days. Complicated cases of malaria are treated parenterally with quinine and referred to a hospital. Treatment for malaria is free at all rural clinics in Gwanda.

### Data collection

Data from the District Health Information System (DHIS) and the health facilities in Buvuma, Ntalale and Selonga were collected retrospectively for the period 2005–2015 to assess trends in malaria incidence. The DHIS is an electronic database of all reported health conditions for the district. The DHIS contains aggregated data from health facilities. There is some specific information that can only be obtained from the health facility, such as specific ages of patients and malaria cases by month per facility. In this study, information from both the DHIS and health facility was used as these complemented each other. At each health facility, data from the malaria registers was extracted. Data on all reported malaria cases for the period at ward level was collected. This included both confirmed and unconfirmed cases. A malaria case was defined as any patient that tested positive for malaria during the 2005–2015 period. For each case data on sex and age were collected.

### Climatic variables

Mean monthly maximum and minimum temperatures (land surface temperatures) and monthly precipitation figures for Buvuma, Ntalale and Selonga wards were obtained from remote sensing. For each month, minimum, maximum and average temperatures were calculated. The mean average temperature was calculated as the mean of the average monthly temperatures. global positioning system (GPS) coordinates were used to define ward boundaries Precipitation data for each ward was collected from climate hazards group infrared precipitation with station data (CHIRPS) database [29] while land surface temperature (LST) data was obtained from moderate resolution imaging spectro-radiometer (MODIS) satellite data [30, 31]. Climatic variables data were collected from satellites because they have been shown to have an even spatial distribution [32]. The spatial resolution for MODIS and CHIRPS was 1 and 5 km respectively.

### Data analysis

Data analysed using SPSS 22.0 (SPSS Inc., Chicago, IL) and R version 3.2.4 (Team RC. R: A language and environment for statistical computing. R Foundation for Statistical Computing, Vienna, Austria. 2017). Graphs to describe the trends of malaria incidence over the study period were generated. Time series correlograms stratified by ward to determine seasonality of malaria transmission in the three study wards were used. A linear regression was performed to assess the significance of the malaria incidence trends over time. The malaria incidence rates were correlated to climatic variables LST (°C) and precipitation (mm). LST was measured as mean minimum, mean maximum and mean average temperatures for each month. Pearson correlation coefficients were calculated to determine the association between malaria incidence and the climatic variables. The climatic variables were lagged at 0- to 12-month lag period to determine the maximum significant positive correlations.

As a sensitivity analysis, the correlation coefficients were estimated using the Spearman rank correlation coefficient (non-parametric equivalent which is less sensitive to distributional assumptions). The distributed lag non-linear models (DLNM) to was employed to assess the temporal lagged effect of monthly climatic variables on monthly malaria incidence. This approach has been employed previously to assess the temporal lagged association between the variables concerned [33–35]. A quasi-Poisson distribution was assumed to allow for over dispersion given the observed distribution of monthly incidence.

The excessive number of months with zero malaria case counts does present a problem for conventional modelling approaches. While the quasi-Poisson approach which was employed may account for dispersion issues, it may not sufficiently account for excess zero process observed in these data. Two problems exist in this data, which may preclude the use of a standard Poisson modelling approach to relate climate variables with malaria incidence. Firstly, if there is over dispersion and secondly, if the data exhibit excessive zero counts that is not suitable for the Poisson approach (as is the case in these data). The hurdle model is one class of model capable of capturing both properties [36–38]. The model comprises two-components. Firstly, a truncated count component, such as Poisson or negative binomial, is employed for positive counts, while secondly a hurdle component models zero vs. larger counts. For the latter component, a binomial model or a censored count distribution are commonly employed. The hurdle model in this analysis was tested by employing a negative binomial and binomial process for the two respective aforementioned components. The model output from the quasi-Poisson approach was compared with the hurdle model and significant differences were not observed.

Lags 0–12 months were modelled for each of the climatic explanatory variables. For each climatic variable a natural cubic spline basis (with 3 degrees of freedom) was employed to try and capture the non-linear effects and their lag dimensions. The models were implemented in R using the “dlnm” and “splines” packages.

## Results

### Malaria trends

**Fig. 2:**
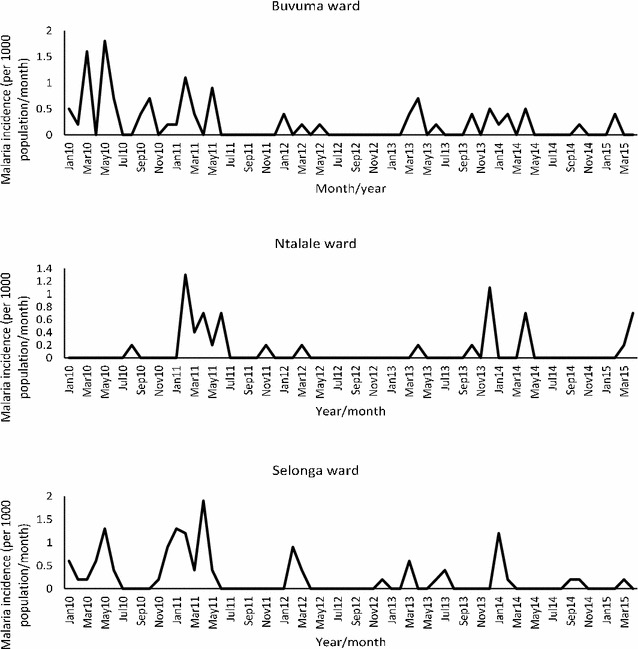
Trends of incidence rates for confirmed malaria cases reported for Buvuma, Ntalale and Selonga wards for the period January 2010 to April 2015

Most malaria cases were reported during the rainfall season (November to May), with major malaria incidence peaks occurring around the month of April. There were 246 confirmed malaria cases in the three wards with Buvuma, Ntalale and Selonga contributing 30, 19, 51% of the cases, respectively, for the period 2005 to 2015. Overall, the highest incidence rate (1.9/1000 population) was reported in Selonga in 2011. Mean monthly malaria incidence varied from 0 to a maximum of 1.9/1000 population across the three wards (Fig. 1). The majority of malaria cases (95%) occurred in the > 5 year age category. Temporal trends in malaria incidence for Buvuma and Selonga showed almost similar patterns with the highest peaks in 2011 and 2013. There were no reported cases of malaria in Ntalale for the period 2005–2008 resulting in the ward having the lowest number of malaria cases over the study period. However, there was a sharp increase in incidence in 2011 with a peak of 1.3/1000 population in February in that ward. After 2013, all three wards experience a general decrease in malaria incidence compared to the previous period. The trends shown in Fig. 2 are for the period 2010–2015 only as data for the previous years was not available for some of the months.

When trends of malaria incidence were analysed using a linear regression, the reduction in malaria incidence over the period 2005–2015 was statistically significant for Buvuma (*F* = 9.21, p = 0.004) and Selonga (*F* = 8.57, p = 0.005) wards. However, the same trends were not statistically significant for Ntalale ward.

### Trends of confirmed and unconfirmed malaria cases

**Fig. 3:**
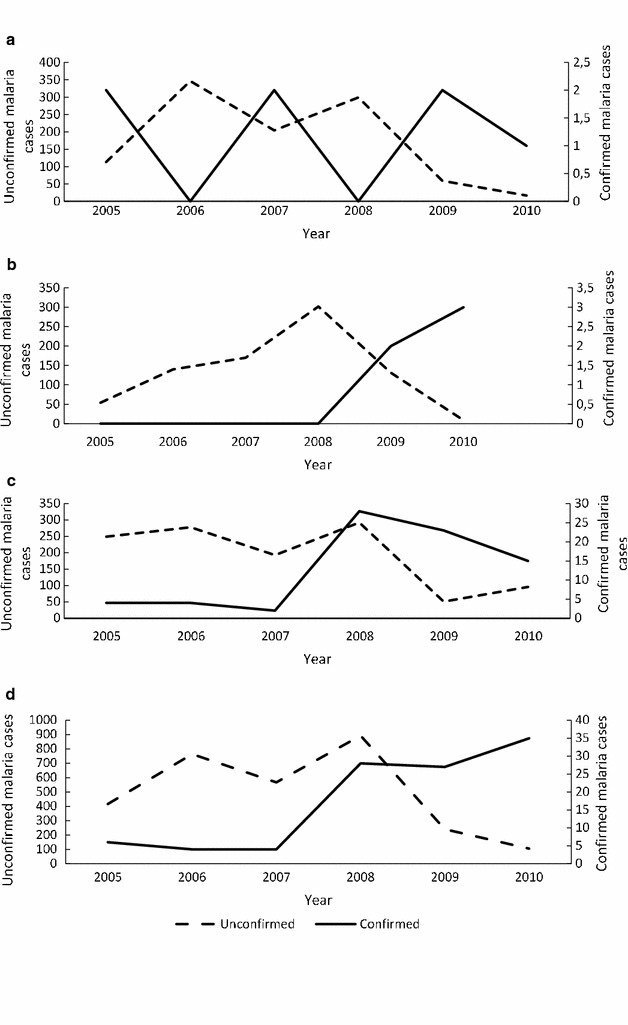
Comparison between the number of confirmed and unconfirmed malaria cases for Buvuma (graph A), Ntalale (graph B), Selonga (graph C) and combined wards (graph D) for the period 2010–2015

The results of this study showed no correlation between clinical malaria (unconfirmed) cases and confirmed malaria cases in all the three wards studied based on available data covering 2005–2010 (Fig. 3). The number of confirmed malaria cases was 0.67, 0.62 and 6% of all suspected (unconfirmed) malaria cases in Buvuma, Ntalale and Selonga respectively.

### Precipitation and temperature trends

The mean monthly climatic variables for the three study wards (Table 1) showed that Ntalale ward had high mean monthly precipitation during the study period compared to Buvuma and Selonga. All the other variables were very closely related across the wards. The combined mean minimum, mean maximum and mean average temperatures for the three wards were 19.02, 40.09 and 29.55 °C, respectively.

**Table 1:** Mean monthly climatic variables for Buvuma, Ntalale and Selonga wards for the period 2005–2015

Climatic variable	Ward			
	Buvuma (mm) (range)	Ntalale (°C) (range)	Selonga (°C) (range)	Overall (°C) (range)
Mean precipitation	27.0 (0.4–160.8)	33.5 (0.6–197.9)	29.3 (0.8–179.4)	29.9 (0.4–197.9)
Mean minimum temperature	19.2 (7.3–22.0)	19.2 (7.6–22.0)	18.6 (6.5–21.3)	19.0 (6.5–22.0)
Mean maximum temperature	41.3 (30.8–52.7)	38.9 (29.3–49.9)	40.1 (30.7–51.6)	40.1 (29.3–52.7)
Mean average temperature	30.3 (19.6–37.1)	29.4 (19.2–36.0)	29.0 (19.2–36.3)	29.6 (19.2–37.1)

**Fig. 4:**
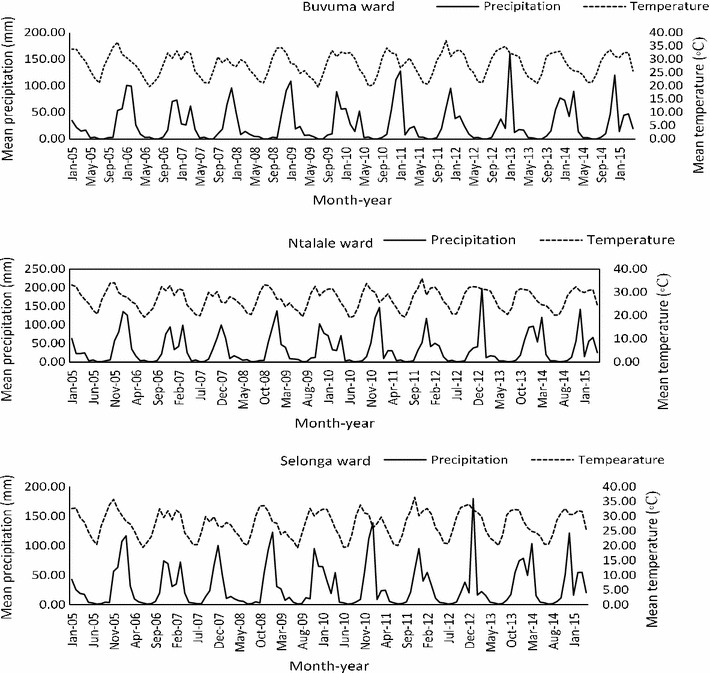
Trends for mean monthly precipitation and temperature for Buvuma, Ntalale and Selonga wards for the period 2005–2015

The monthly precipitation peaked between November and January for all the three study wards (Fig. 4). The precipitation trends followed the same general pattern with Selonga having slightly higher precipitation peaks compared to Buvuma and Ntalale. The highest peak for precipitation was in January 2013 for all the three wards. Notably, one of the three main peaks in malaria incidence during the 2005–2015 period was also in 2013. Mean minimum, mean maximum and mean average temperatures for the three wards showed slight variations. The mean monthly temperatures showed a fluctuating trend from 2005 to 2015 (Fig. 4). The highest mean temperature was recorded in 2011 (37.13 °C) in Buvuma ward. Overall, Buvuma ward recorded slightly higher temperatures than Ntalale and Selonga.

### Relationship between malaria incidence and climatic variables

There was no significant correlation between malaria incidence and climatic variables at 0-month lag period except for mean minimum temperature in Selonga ward. There was a significant correlation between malaria incidence and the climatic variables in Buvuma and Selonga wards at specific lag periods (Table 2). Precipitation was significantly and maximally correlated with malaria incidence in all the three study wards at 1-month lag period. In Ntalale ward, only precipitation (1- and 3-month lag) and mean temperature (1- and 2-month lag) were significantly correlated with incidence. There was also no significant correlation between incidence and climatic variables in all the three wards at 6-month lag period. The maximum positive and significant correlation between malaria incidence and mean minimum temperature occurred at 2-month lag period in all the three wards. Overall, the maximum positive correlation between incidence and minimum temperature occurred at 2-month lag in Selonga (r = 0.365). Beyond the 6-month lag period, there were no significant positive correlation between malaria incidence and climatic variables.

**Table 2:** Association between malaria incidence and lagged climatic variables measured by the Pearson’s correlation coefficient

Lag period	Buvuma				Ntalale				Selonga			
	Precipitation	Min. Temp.	Max. Temp.	Ave. Temp.	Precipitation	Min. Temp.	Max. Temp.	Ave. Temp.	Precipitation	Min. Temp.	Max. Temp.	Ave. Temp.
Unlagged	− 0.0692	0.1144	− 0.0911	− 0.0003	0.0004	0.0803	− 0.0672	− 0.0007	0.1673	0.2131*	− 0.1736	0.0037
1-month lag	0.2375*	0.2301*	− 0.0715	0.0662	0.2105*	0.1815*	− 0.0418	0.0665	0.2513*	0.3543*	− 0.0036	0.1802
2-month lag	0.2817*	0.2748*	0.0659	0.1758	0.1272	0.1864*	0.019	0.1062	0.2833*	0.3656*	0.1191	0.2580*
3-month lag	0.1703	0.2651*	0.1792	0.2400*	0.2009*	0.1816	0.0264	0.1079	0.2427*	0.3507*	0.2674*	0.3400*
4-month lag	0.1689	0.1854	0.2204*	0.2231*	0.1557	0.1245	0.1054	0.127	0.1251	0.2275*	0.3455*	0.3252*
5-month lag	0.0763	0.1179	0.2329*	0.1968*	0.0584	0.0556	0.093	0.0848	− 0.008	0.032	0.1743	0.1229
6-month lag	0.0096	− 0.0561	0.0931	0.0297	− 0.1012	− 0.0362	0.1446	0.0699	− 0.0995	− 0.1664	0.0204	− 0.0707

Italic: maximum positive correlation

* Significant correlation at the 5% level of significance

**Fig. 5:**
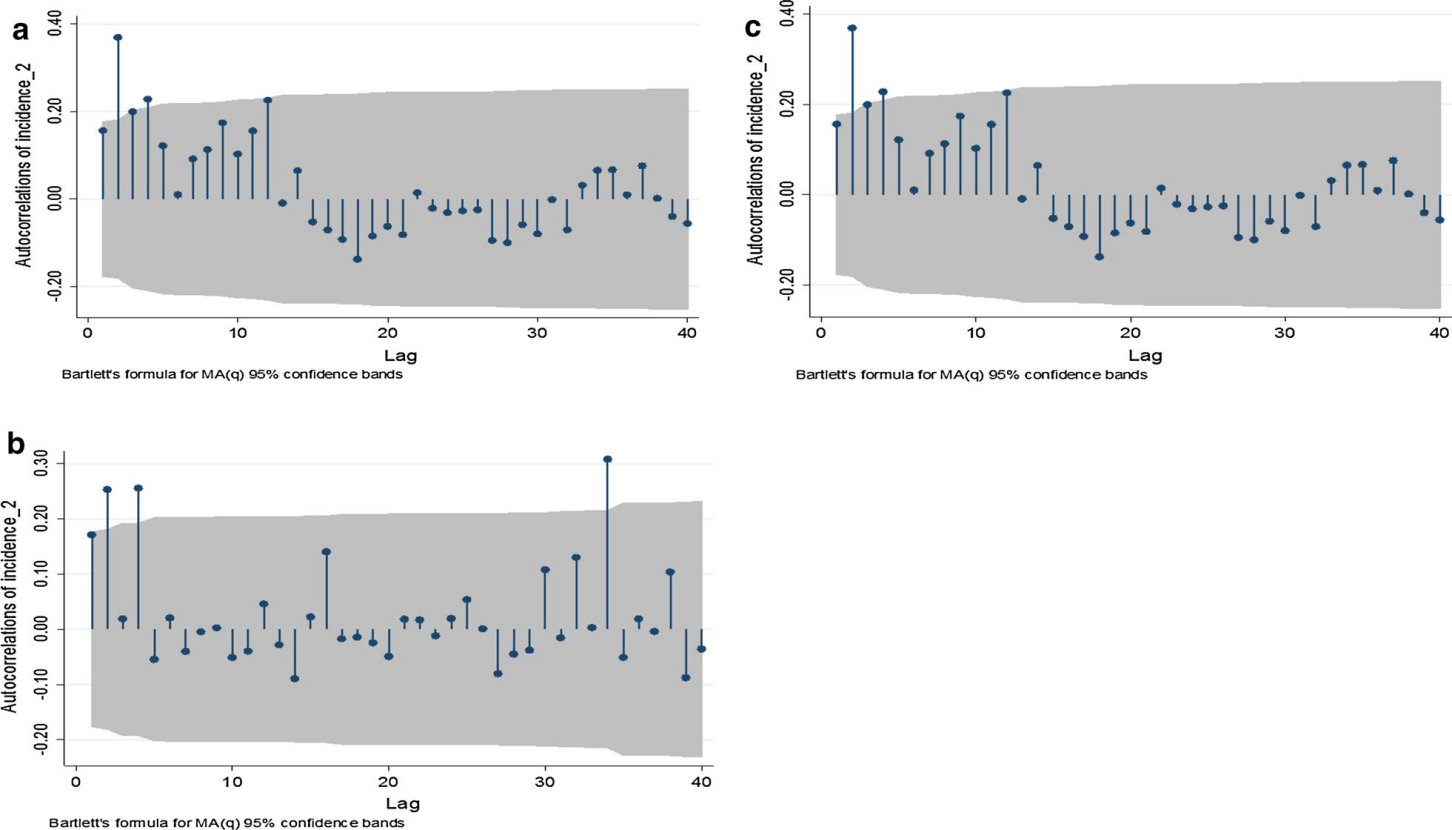
Time series correlograms stratified by ward for Buvuma (**a**), Ntalale (**b**) and Selonga (**c**) wards

A sensitivity analysis was performed to estimate the correlation coefficients using the Spearman rank correlation. This analysis gave similar results to the Pearson correlation coefficient. Use of time series correlograms (autocorrelation plots) stratified by ward suggests 12-month seasonality in Buvuma and Selonga but not in Ntalale, the latter being more sporadic (Fig. 5). Additional analysis highlights how the transmission of malaria is linked to seasonal climatic variables (by assessment of lag and distribution of correlation over a 12 month season).

**Fig. 6:**
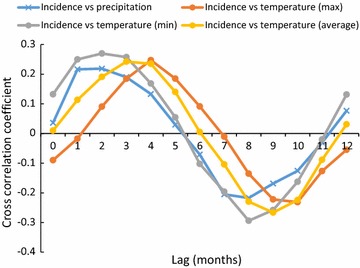
Cross-correlation coefficients of time series on monthly climatic variables (precipitation and temperature) and monthly malaria incidence at various lag periods. The grey shaded area represents the 95% confidence band for the autocorrelations. Blue dots represent autocorrelations of the lagged variables

Overall, cross-correlation coefficients of time series of monthly climatic variables and monthly malaria incidence showed that the minimum monthly temperature at 2-month lag was a positive and strong predictor for an increase in malaria incidence (Fig. 6).

**Fig. 7:**
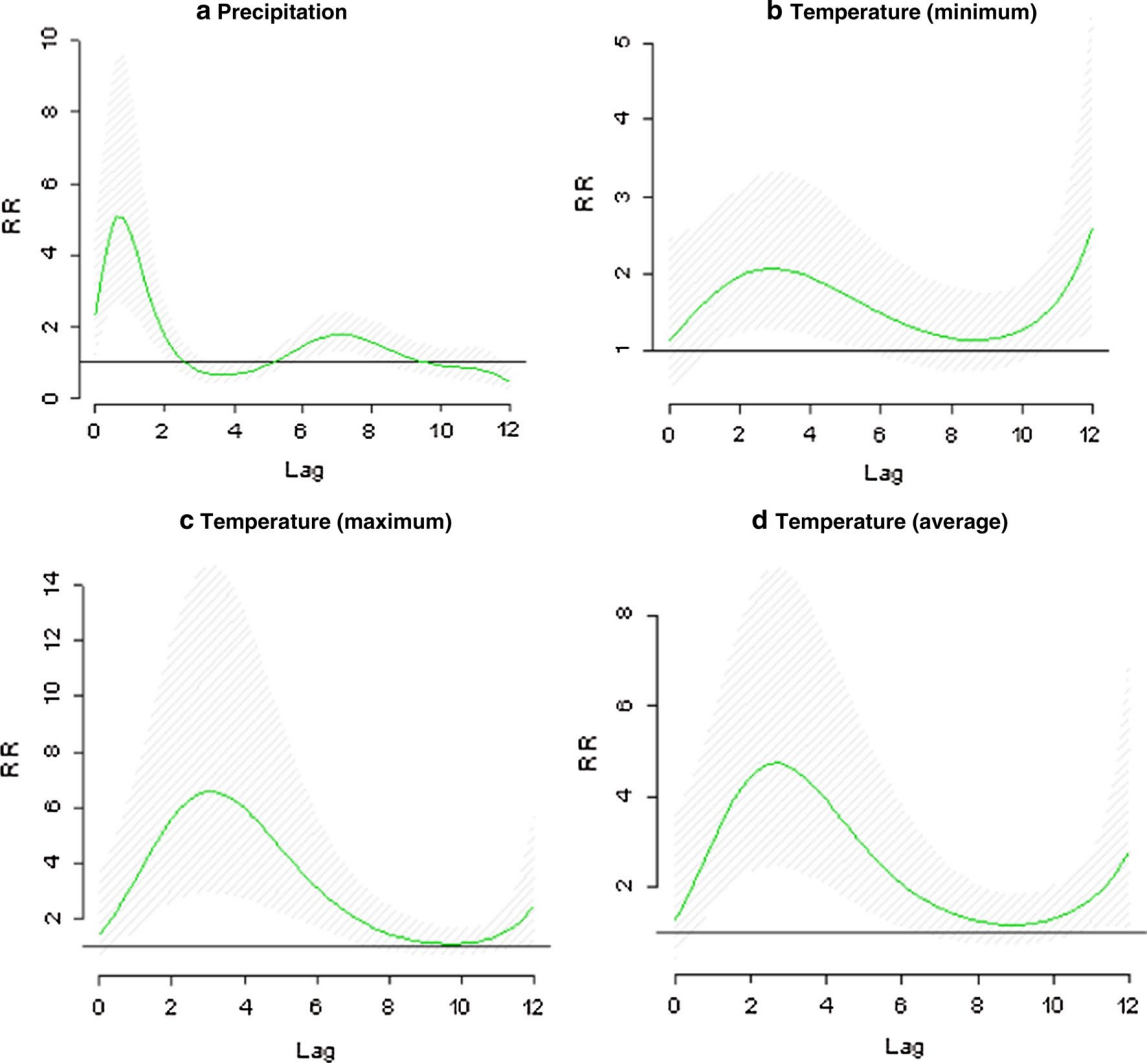
Distributed lag between precipitation, minimum temperature, maximum temperature and average temperature. The green line is the estimated distribution lag (month), while the shaded bands indicate the 95% confidence interval. The horizontal axis shows the relative risk (RR)

The DLNM to assess the temporal lagged correlation between monthly malaria incidence and monthly climatic variables supported the optimal lag periods identified using the lagged cross-correlation coefficient analysis. The distributed lag curves between malaria incidence and the climatic variables is shown in Fig. 7. The model results suggest a strong and significant increase in monthly malaria incidence relative risk at lags 0 to − 2 for precipitation with peak at lag − 1 month. The association between lagged minimum temperature and monthly incidence occurred at lags – 2 to 4 months with a peak at − 3 months. Similarly association was observed for maximum and average temperatures though the strength of association was not as strong as observed for the preceding two climatic indicators (Fig. 7).

## Discussion

Data from most malaria surveys in Zimbabwe are aggregated at national or district levels and do not give detailed patterns of malaria at lower levels such as the ward. Our study used ward level data in a malaria endemic rural area of Zimbabwe. The study was based on confirmed cases of malaria and therefore gives a more accurate picture of the malaria situation in the area as opposed to using data from unconfirmed cases of malaria as done in some previous studies [39–42].

Results from this study shows that the preceding 1–4 months of climate is more correlated with current malaria incidence. Climatic variables at 1–4 months lag period can, therefore, be considered as early-warning indicators and can play a role in planning for malaria control. Most significant correlations were observed between minimum temperature (range 6.5–22.0 °C) and malaria incidence compared to average (range 19.2–37.1 °C) and maximum temperatures (range 29.3–52.7 °C). The minimum temperature was significantly associated with malaria incidence at 1- and 2-month lag periods in all the study wards. This seems to suggest that minimum temperatures have a greater influence on malaria transmission in the study area compared to higher temperatures. Similar observations were made in studies in Botswana [42] and Rwanda [43].

The results from this study show that malaria incidence is significantly associated with precipitation in all the three wards at 1-month lag period. This shows that precipitation can play an important role in malaria transmission in the study area at 1-month lag. The findings of this study on the influence of precipitation on malaria incidence were different from previous studies which showed that higher rainfall does not necessarily result in significant changes in malaria cases [44, 45]. Results from different studies on the relationship between climatic variables and malaria are quite varied, thereby indicating that this relationship is complex [46].

The results suggest 12-month seasonality of malaria transmission only in Buvuma and Selonga, but not in Ntalale. In order to explain this difference, there is need to study the role of other variables such as timing and types of malaria control activities specific to each ward. These variables were not included in this present study because of the unavailability of complete data on malaria interventions at the time of the study.

Several studies have shown the effect of climatic variability on malaria transmission [12–19, 32]. A study on climate and vector borne disease by Mondzozo et al. [15], showed that Zimbabwe’s average annual changes of malaria cases per 1000 as a result of climate change was 98 cases between the years 1990 and 2000. That same study also showed that an estimated 1% increase in temperature per annum led to 12.65 more malaria cases, while an estimated 1% change in precipitation led to a − 0.53 change in malaria cases. Modelling has shown that optimal malaria transmission occurs at 25 °C and malaria transmission decreases at temperature above 28 °C [47]. Temperatures below 16 °C are also detrimental for survival of mosquitoes [48]. Maximum temperatures have been shown to frequently approach or exceed the upper limits of mosquito survival [49]. The mean minimum, mean maximum and mean average temperatures for the study area were 19, 40 and 29 °C, respectively. This indicates that the mean minimum temperature may have been in the range for survival of mosquitoes. However, the mean maximum and average annual temperature were outside the optimal range of temperature for malaria survival. Results from a previous study showed a strong positive association between malaria incidence in a given month and the minimum temperature of the previous month. This indicates that the minimum temperature is an influential climatic variable for malaria transmission [50].

The study showed that malaria incidences are higher (95%) in the > 5 years age category. Malaria control interventions in the country have mostly been targeted at pregnant women and children below 5 years as they are the most vulnerable. This has resultantly led to a decrease in incidence in these two social groups. This could possibly explain why our results show less infections in the below 5 years age category. Studies from Botswana showed similar findings [42, 51] in contrast to studies elsewhere [52]. A higher burden of malaria in older ages has economic implications, as the age group is the most economically productive. It is therefore critical for malaria control interventions to target all age categories for better impact. It has previously been reported in some studies that in areas of high transmission, the malaria burden mainly among children and infants, while in areas of low transmission, older ages carry the most burden [25, 52–54]. The results of this study concur with these previous findings.

Contrary to results from a previous study [55], we did not find a correlation between the trend of confirmed and unconfirmed (clinical) cases of malaria. This suggests that that the trend of clinical malaria cases may not be reliably used to represent the trend of confirmed cases. Thus, it is important to assess the trend of malaria using confirmed cases as opposed to clinical cases as the latter does not provide a true picture of the trend. It was noted that in all the study wards, the number of confirmed malaria cases was less than 10% of all suspected cases. Unconfirmed (suspected) cases of malaria are those that would have presented to the health facilities with fever. These fevers could be an indication of other health problems in the community that result in fevers [56]. A previous study showed that in Zimbabwe, clinical diagnosis over-estimates malaria cases by 73% [40]. Over-diagnosis of malaria will lead to failure to treat the alternative causes of severe infection [41] and may lead to drug resistance through treatment of uninfected people. Before 2011, all suspected malaria cases were treated for malaria since the rapid diagnostic tests (RDT) were not yet available at health facilities in the district. However, from 2011, the policy has been to test all suspected malaria cases with RDT thereby ensuring that only positive cases are treated. This new malaria treatment policy is, therefore, important in curbing drug resistance due to treatment of malaria negative patients.

This study had some limitations. Secondary data from health facilities was used and, therefore, there was a possibility of missing or incomplete data from some of the records. Health facility data has a potential for under-reporting malaria cases, as some may not have presented at the health facilities. Health facility-based data can be affected by many factors including accessibility of the facilities by patients, perceptions of care and whether or not self-treatment is available to the patients [57]. The other limitation was that some confounding factors might have influenced the results of the study. These include the impact of malaria control activities and other related interventions.

## Conclusions

Data from the health information system were utilized to determine the trends of malaria incidence and their relationship with climatic variables at ward level in a rural community. The study found that precipitation and temperature play a role in influencing malaria incidence at specific lag periods and can, therefore, assist in prediction of malaria risk. As the period of high malaria risk is associated with precipitation and temperature at 1–4 month prior in a seasonal cycle, intensifying malaria control activities over this period will likely contribute to lowering the seasonal malaria incidence. Further monitoring of malaria incidence in affected communities has the potential to reveal future trends of malaria transmission thereby leading to improved control strategies.

## Abbreviations

°C: degree celsius
%: percent
CHIRPS: climate hazards group infrared precipitation with station
DHIS: district health information system
MODIS: moderate resolution imaging spectro-radiometer
RDT: rapid diagnostic test
